# Unmet Medical Needs and Early Referral of Pediatric Atopic Dermatitis: An Expert Modified Delphi Consensus from Saudi Arabia

**DOI:** 10.1155/2022/5636903

**Published:** 2022-08-11

**Authors:** A. Alradaddi, A. Al Twaim, A. Abu-aliat, K. Al-Atass, L. Alogayell, M. Aldayil, S. AlBreiki, S. Abed, M. Fatani, O. Alsharif, B. Darwesh, Levent M. Gunay, S. Al-Khenaizan

**Affiliations:** ^1^National Guard Hospital, King Abdulaziz Medical City, P.O. Box 9515, Jeddah 21423, Saudi Arabia; ^2^College of Medicine, King Saud Bin Abdulaziz University for Health Sciences (KSAU-HS), Jeddah, Saudi Arabia; ^3^King Saud Bin Abdulaziz University for Health Sciences, Riyadh, Saudi Arabia; ^4^Department of Dermatology, Armed Forces Hospital Southern Region, Tamniah, Saudi Arabia; ^5^King Fahd Central Hospital, P.O. Box 204, Jazan, Saudi Arabia; ^6^King Fahad Medical City, Riyadh, Saudi Arabia; ^7^Dermatology Department, E1 Cluster, Dammam, Ministry of Health, Saudi Arabia; ^8^King Fahad Hospital, Khobar, Saudi Arabia; ^9^Research and Development, King Abdullah International Medical Research Center (KAIMRC)-Western Region, Jeddah, Saudi Arabia; ^10^Dermatology Department, Hera General Hospital, Makkah, Saudi Arabia; ^11^Pediatric Dermatology, King Fahad General Hospital, Medinah, Saudi Arabia; ^12^Pfizer Saudi Limited, Jeddah, Saudi Arabia; ^13^Pfizer, İstanbul, Turkey; ^14^King AbdulAziz Medical City, Riyadh, Saudi Arabia

## Abstract

Atopic dermatitis (AD) is a chronic skin disease with increasing prevalence worldwide. It is characterized by pruritic eczematous lesions, affecting up to 20% of the children and negatively impacting their quality of life. Guidelines for AD management are available worldwide, but specific guidelines for pediatric AD in Saudi Arabia are lacking. This consensus document aims to identify the needs for the diagnosis and management of pediatric AD in Saudi Arabia by gathering the opinions and recommendations of key experts. We conducted a three-step modified Delphi method to develop the present consensus. The experts agreed that pediatricians and dermatologists commonly encounter AD; however, it is still under-recognized in its early stage in Saudi Arabia. The family physicians should be involved in assessing suspected children with a family history of atopy, particularly in patients with isolated lesions. Further, the experts confirmed that AD diagnosis should be documented, showing assessment criteria used, key morphological characteristics, and features used to ascertain the severity of the disease. There is still a need for simple validated diagnostic criteria suitable for daily practice for pediatric AD. The experts highlighted several medical conditions that pertain to the diagnosis and management of AD in Saudi Arabia.

## 1. Introduction

Atopic dermatitis (AD) is a chronic, relapsing-remitting skin disorder, affecting up to 20% of children and 5–10% of adults [1–3]. The disorder presents with intense itching and recurrent eczematous lesions, often affecting patients with atopy, including allergic rhinitis or asthma, and results in significant morbidity and worsening quality of life (QoL) [4, 5]. The pathophysiology of AD is complex and multifactorial, including genetic causes, epidermal barrier defects, altered immune responses, microbial colonization, and lifestyle and environmental factors [6].

Pediatric AD is a common skin disease, commonly presented during the first 6 months of age [7]. Approximately 60% of pediatric patients have AD symptoms during the first one year of age [8] and about 85% by the age of 5 [9]. Pediatric patients with AD might have disease progression or enter remission; however, the disease commonly persists into adulthood [10]. In patients with obvious remission, they maintain abnormal skin and tend to develop clinical manifestations [11]. A cohort study reported that 50% of children affected by AD persisted into adulthood and correlated with early onset of the disease, allergic rhinitis during childhood, and hand eczema [12].

The objective of AD management is to restore skin barrier function, regulate abnormal immune responses, and improve symptoms and signs in the short- and long-term [13]. Topical corticosteroids and other topical agents are considered first-line therapy in mild-to-moderate patients [14]. When topical agents fail, further conventional systemic treatments, including cyclosporin, methotrexate, azathioprine, and mycophenolate mofetil can be added [15]. Targeted therapy with a dupilumab monoclonal antibody is indicated for the treatment of moderate-to-severe AD in adults and adolescents 12 years and older, and for treating severe atopic dermatitis in children 6–11 years old, who are candidates for systemic therapy [16].

Although the pathogenesis of AD is well recognized, the consensus on daily management practice is limited [17, 18]. Available recommendations are focused on adult AD; therefore, an expert panel consisting of dermatologists and pediatricians from Saudi Arabia was convened to develop a consensus on the unmet needs for the diagnosis and management of pediatric AD and the importance of proper referral guidance. This expert panel meeting aimed primarily to bring together practical treatment recommendations and provide an easy guide for driving therapy and management in daily clinical practice.

## 2. Methods

### 2.1. Study Design

The development of the present consensus was based on a three-step modified Delphi method, which involved two rounds of voting and a virtual meeting. A total of 11 pediatricians, pediatric dermatologists, dermatologists, and experts from Saudi Arabia were recruited through a purposive sampling technique. Experts were affiliated with educational institutions or the Ministry of Health (MoH) from the Kingdom and had active research profiles in the field of AD.

### 2.2. Survey Development and Voting

An online search was conducted on Medline via PubMed to collect relevant information on AD diagnosis, severity assessment, management approaches, education and research, and patients' journey and referral. The literature search included the key terms atopic dermatitis, pediatric atopic dermatitis, atopic eczema, Saudi Arabia, consensus, expert opinion, recommendations, guidelines, treatment, and diagnosis. The statements were primarily extracted from studies with level 1 quality of evidence, as classified by Wright et al. [19]. A total of 46 consensus statements were developed based on five main areas: (i) the diagnosis and severity assessment of AD, (ii) AD treatment, (iii) education and research of AD, (iv) the impact of AD on caregivers, and (v) patient journey and criteria for early referral. Each statement was considered a consensus if it achieved an agreement level of ≥75% [20]. The statements, which did not reach the agreement level, were persevered for step two to be modified or omitted by the experts.

## 3. Results

The list of the statements with the agreement percentage is reported in Table 1. At the 1st voting round, most statements had achieved 100% consensus, except some of them had obtained a rather low level of consensus. Following discussion and amendments introduced at the meeting, almost all statements reached a higher level of consensus. Each approved final statement is presented and discussed hereafter.

## 4. Result and Discussion

This consensus study aimed to identify the current needs in diagnosing, assessing, and managing pediatric AD. High agreement percentages for all statements were achieved, suggesting that the process used was successful in attaining consensus on different topics related to the diagnosis and management of pediatric AD.

### 4.1. The Diagnosis and Severity Assessment of AD

The experts confirmed that AD is commonly encountered by pediatricians and dermatologists; nonetheless, it is still under-recognized in its early stage in Saudi Arabia. AD patients prefer medical consultations by specialists rather than a family medicine physician or a general practitioner. This could be one of the factors that result in significant waiting lists and potentially delayed diagnosis and proper management [21]. In addition, family physicians in Saudi Arabia showed several misconceptions when dealing with dermatological conditions, including fear of prescribing topical corticosteroids, according to a survey in Jeddah. This was driven by the fact that undergraduate education was their only source of dermatology knowledge [22]. Hence, the panel of experts recommended that family physicians be involved in assessing suspected children with a family history of atopy, particularly in patients with isolated lesions, since in Saudi Arabia, family practitioners are the ones who usually are consulted for initial diagnosis and treatment [23, 24].

This statement is supported by previous cross-sectional studies that showed that 26% out of a total of 243 children had atopic dermatitis [25]. Another study published in 2019, which included children from six schools in Eastern Saudi Arabia, showed that the prevalence of skin disorders among these children was 23.6%, of which 11 (11.3%) had allergic dermatoses and 14 (11.8%) had eczema [26]. Another single-center study in Jeddah showed that 41.7% of the children attending the University's dermatology clinic in 2017 had AD [27]. On the other hand, a cross-sectional study involving 1,337 male children in Al Hassa reported a total of 47 (3.5%) children affected by eczematous lesions [28].

The experts agreed that AD diagnosis should be documented, showing the criteria used, key morphological characteristics, and features used to ascertain the severity of the disease. Nonetheless, there is still a need for simple validated diagnostic criteria for pediatric AD suitable for daily practice. AD diagnosis is mainly clinical. It is not usually challenging in children with chronic relapsing-remitting eczema in typical body areas or adults with similar lesions and a childhood history of AD. In addition, severity evaluation of AD should depend on healthcare provider assessment, given no assessment method adequately detects this in clinical practice [29]. Moreover, the cause and morphology of lesions can be changed with time. Indeed, AD diagnosis is often by exclusion, although additional diagnostic tests are usually needed, such as patch testing. It should be recognized that chronic hand eczema can be related to AD or may represent comorbidity of the condition [30].

AD diagnosis can be based on the patient or family history of atopies such as asthma or allergic rhinitis. Food allergy associated with AD is frequently found in children younger than 5 years. Thus, a food allergy test may be undertaken in patients of moderate or severe AD patients who respond poorly after appropriate treatment [31]. Thus, an oral food challenge test should be performed on children to confirm the diagnosis of food-induced eczema in patients with AD to avoid unnecessary food avoidance [24, 32].

The experts agreed that the use of severity scales and QoL scale is under-utilized in routine Saudi practice, owing to their complexity and time-consuming nature. Clinicians should examine the impact of disease on QoL during clinical visits. The experts confirmed that there is a need for validated Arabia patient-reported outcomes to measure the impact of AD on the QoL of the patients and their parents/caregivers. Alzolibani investigated the QoL among patients with AD in Saudi Arabia, using the Arabic version of the infants' dermatitis quality of life (IDQoL) index. The study showed that AD symptoms impaired the IDQoL of patients and were correlated with the disease severity score. Itching, mood, and time to get the child to sleep had a higher negative effect on IDQoL [33]. Another study by Alzolibani showed higher IDQoL scores among AD infants compared with their respective controls (*P*=0.00) [34]. A cross-sectional study by Abolfotouh et al., including 283 adults with skin diseases, showed that the QoL was good in 69% of the participants. The emotional domain was the most affected (mean score 44.27 ± 27.06), then symptoms (31.45 ± 28.40) and functioning (14.61 ± 22.75). After adjustment for potential confounders, poorer QoL was significantly associated with female gender (*P*=0.03), older age (*P*=0.003), rural origin (*P*=0.03), positive family history of the same lesion(s) (*P*=0.01), shorter duration of ≤6 months (*P*=0.02), generalized spread (*P* ≤ 0.02), and lack of isotretinoin treatment (*P*=0.02) [35].

Although laboratory tests and biomarkers can be used initially to diagnose AD, there is no need for biomarkers for assessing the severity of AD. There are two subtypes of AD, intrinsic and extrinsic. Normal total IgE levels were shown in patients with an intrinsic form without specific IgE. However, high total IgE levels were found in patients with an extrinsic form of AD and are often sensitized to multiple allergens [36]. Therefore, total serum IgE can be used initially as a diagnostic biomarker for AD patients. Many biomarkers have been investigated as a marker for disease severity, such as serum thymus and activation-regulated chemokine [37]. An Italian consensus stated that a list of biomarkers and tests would be important in the diagnosis of AD [38], especially in difficult-to-diagnose patients [39].

The experts agreed that although AD is primarily a clinical diagnosis, many unrequired tests are usually ordered by the healthcare providers in the Saudi setting, despite the lack of sufficient data to support their diagnostic and/or prognostic utility. The development and utilization of severity scores, with validated thresholds for treatment choice, are critical steps as the treatment's decision is mainly based on disease severity. Assessment of disease severity is vital regarding treatment planning and further investigations [40]. Several international scores have been used for assessing the severity of AD, such as Eczema Area and Severity Index (EASI) [41], Scoring Atopic Dermatitis (SCORAD) [42], and Peak Pruritus Numerical Rating Scale [43]. In 1980, Hanifin and Rajka developed criteria for a diagnosis of AD presented in Table 2. Three out of four major criteria and 3 out of 23 minor criteria are needed for AD diagnosis [44].

### 4.2. AD Treatment

**Figure 1:**
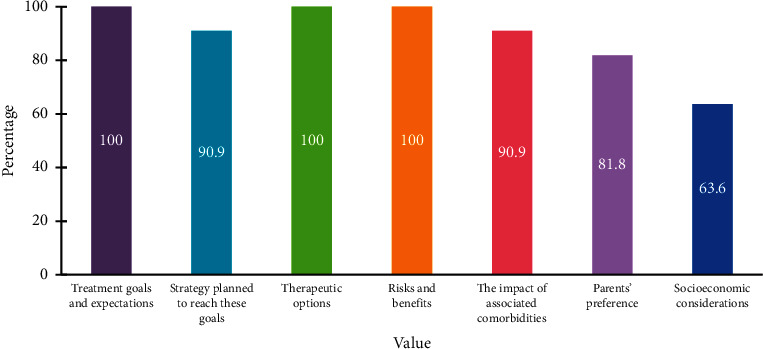
Factors that should be considered in the shared decision-making regarding the treatment of AD.

**Figure 2:**
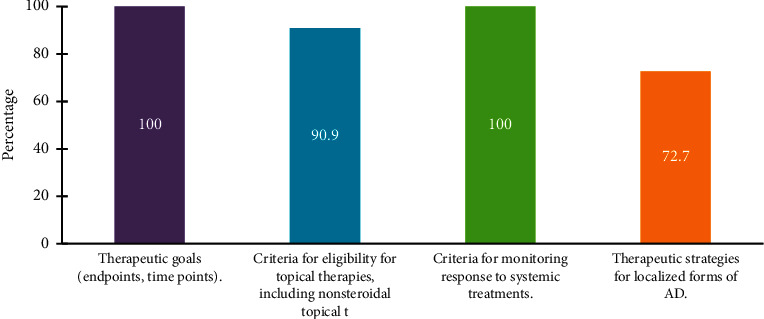
Factors that should be incorporated in therapeutic protocols of AD.

The experts agreed that there is a discrepancy between dermatologists, pediatricians, allergists, immunologists, and family physicians in managing atopic dermatitis in children. Further, the treatment of pediatric AD should be based on shared decision-making between the parents, caregivers, and provider. The goals of such treatment are to reduce symptoms, including pruritus and dermatitis, decrease exacerbations, and avoid therapeutic risks. The shared decision-making should involve treatment goals and expectations, strategy to reach these goals, therapeutic options, risks and benefits, the impact of associated comorbidities, and parent's preference (Figure 1). The expert panel agreed that therapeutic protocols are still lacking in Saudi Arabia. These protocols should incorporate therapeutic goals (endpoints, time points), criteria for eligibility for topical therapies, including nonsteroidal topical therapies, and criteria for monitoring response to systemic treatments [45] (Figure 2). A long-term curative strategy including patient education, trigger avoidance, proper skincare, and compliance to pharmacologic therapies and nonpharmacologic measures is essential [46].

Reda et al. developed a treatment guideline for AD in adults and children in the Middle East region [47]. The guideline recommended applying emollients on the entire body for all patients with AD to enhance and protect the barrier function of the skin. In patients with acute mild-to-moderate AD in a sensitive body area, the authors suggest applying topical calcineurin inhibitor (TCI), twice daily. However, topical corticosteroids (TCS) may be used for a few days before applying a TCI in some moderate patients. In acute severe AD, TCS should be used for a few days. Once improvement occurs, it is recommended to switch to pimecrolimus to be used on sensitive skin regions or TCIs on other skin regions until a complete resolution of lesions occurs. Despite the resolution of symptoms and signs, continuous application of TCIs to the previously affected area twice or thrice weekly is recommended to prevent symptoms relapse [47].

TCS are usually prescribed as a first-line treatment in pediatric AD; however, Canadian experts agreed that TCS do not need to be the only first-line treatment used. TCI and phosphodiesterase-4 inhibitors can be considered as first-line treatment in certain patients where corticosteroid use is not appropriate, including sensitive skin regions such as the face and intertriginous areas. TCI should not be used on inflamed skin because burning and stinging may occur [29]. Maintenance therapy and flare prevention in pediatric AD can be achieved with intermittent therapy with TCS or TCI. This is a valuable strategy in pediatric AD patients [29]. Many children with moderate-to-severe AD are not receiving systemic therapy because of a lack of recommendations concerning indication and appropriate timing of systemic treatments. The introduction of systemic therapies is usually delayed, which impacts the response to therapy. Systemic steroids are effective, but they can cause undesirable short- and long-term side effects; therefore, they should be considered with caution and in very limited circumstances for severe exacerbations for a short course. While the decision to initiate systemic therapy can be based on disease severity and response to therapy, it may also be related to impact on QoL and daily function, satisfaction with the treatment regimen, adverse events, intolerance, drug interactions, and poor adherence [48]. It is recommended that clinicians establish the goals of systemic therapy and carefully assess the patient before its initiation as recently highlighted [49].

Systemic steroids offer a limited therapeutic role in the treatment of severe AD, both in children and adults [50–52]. Despite their anti-inflammatory effect, corticosteroids have no direct effect on the restoration of the skin barrier and are associated with significant side effects with long-term use [53]. Further, a relapse often occurs post discontinuation.

Schmitt et al. [50] conducted a controlled trial comparing oral prednisolone and cyclosporin versus placebo in patients on topical therapy. Only 3% of the patients showed improvement with more than 75% concerning SCORAD at 2 and 4 weeks of follow-up after steroids administration. This study was discontinued after the occurrence of significant eczema in the patients taking prednisolone [50].

The Practical Allergy Consensus Group guidelines [54] recommended that patients with acute flares may use a short course of systemic corticosteroids; however, they should not be used for long-term use, specifically in children. Consequently, systemic corticosteroid administration in children is not recommended. Short courses of treatment may be considered in the following conditions: a severe exacerbation with intense itching, a transient period before systemic nonsteroidal immunomodulatory drugs, or the presence of associated comorbidities such as a severe asthma exacerbation [55, 56].

Because of safety concerns, many immunosuppressive treatments (such as azathioprine, cyclosporin, and methotrexate) are not recommended for long-term use in children with AD. Insufficient data exist to make clear recommendations regarding the optimal immunosuppressants dosing or duration. Systemic immunosuppressants represent a valid therapeutic option for severe, widespread, and refractory forms of AD since systemic corticosteroids have a limited role in long-term therapeutic management [57]. Systemic immunosuppressants should be used in patients with severe AD negatively affecting the child's QoL. However, systemic immunosuppressant therapy should only be used in specialized institutions. The number of randomized controlled trials investigating systemic therapies is limited; therefore, comparing the relative efficacy of each treatment is difficult. The literature proposes the use of cyclosporin, but azathioprine and methotrexate are also suggested [56].

The expert panel agreed that the introduction of new biologic therapies will likely allow for improved treatment of pediatric AD and attempt to address the needs of AD treatment in this population. The European Union and United States Food and Drug Administration have approved dupilumab, a human monoclonal antibody, as a first-line treatment for patients aged over 6 years with moderate-to-severe AD whose disease is not effectively regulated with topical prescription therapies or when systemic therapies are not advisable [14].

The experts agreed that a considerable proportion of children with AD in Saudi Arabia do not adequately comply with the prescribed treatment. As most treatments are administrated at home with little hospital services involvement, there remain significant challenges in ensuring optimal treatment compliance. The chronicity and necessity for multiple treatment vehicles may add to the complexity of treatment and barriers to adherence. In Saudi Arabia, parents' concerns over the safety profiles of topical and systemic therapies can lead to compliance issues and treatment delays or restrictions [58].

The current consensus confirms that corticophobia is a real issue in Saudi Arabia and needs to be promptly recognized and overcome by patient education programs. Fear of corticosteroids, known as corticophobia, is a significant problem of clinical relevance, being related to decreased adherence to therapy and treatment failure in both pediatric and adult patients [59–61]. A previous Italian consensus agreed that corticophobia needs prompt recognition and further strategies are needed to overwhelm it [38]. The experts recommended educating the patients and clarification about the use of corticosteroids and their side effects and contraindications.

Other factors that contribute to limited compliance in Saudi Arabia include the financial burden of long-term treatment and limited awareness among parents/caregivers. Various strategies should be examined to improve adherence to topical treatment, such as telemedicine technology and proactive intermittent treatment strategies. Adherence to topical therapies for AD was quite high in surveys of physicians in Southeast Asia, yet adherence to systemic therapies was low [62].

The need for effective interventions to improve long-term adherence to moisturizing creams was also recognized by the Italian consensus [38]. Poor adherence is a complex phenomenon and may be related to several factors [63]. For example, it has been noted that many adolescents change the prescribed treatment regimen and do not always adhere to it [46]. It is important to consider how emotional factors like insecurity, inadequacy, anxiety, and depression affect atopic patients [64]. Additionally, the family is afflicted by both economic stresses related to the cost of treatment and by a significant psychological burden, which are mostly heavy in young and severely affected children, that may affect treatment compliance [65].

Greater focus should be given to monitoring adherence to therapy. Moreover, different types of topical agents may be more suited to one form of AD than another [66]. There are currently several topical treatment options available, and the most appropriate choice for individual patients has the potential to improve adherence to therapy [67]. Written eczema action plans (EAP) may be helpful to increase adherence to treatment and decrease treatment failure [68]. A pictogram action plan can be especially helpful in young children. Treatment response should be regularly monitored and modified based on treatment response, tolerability, and patient satisfaction [29].

### 4.3. Education and Research of AD

The experts agreed that educational measures are critical components of any treatment strategies for AD. These measures should be tailored according to patient- and disease-specific factors. Possible educational tools include using traditional materials, support groups, and mobile apps [69]. The education measures should aim to inform parents/caregivers about symptoms and signs of bacterial infection of AD, including weeping, pustules, crusts, eczema failing to respond to treatment, rapidly worsening eczema, fever, and malaise [70]. Education programs for parents can have a significantly positive effect on disease outcomes [71, 72]. Such programs aim to dissipate misunderstandings concerning therapy options and raise awareness of the importance of control assessment methods [73]. Moreover, information and communication technology (ICT) is a powerful tool that can be leveraged to provide medical information to healthcare providers and patients and promote validated tools for AD severity and control [74, 75]. In the era of the COVID-19 pandemic, the use of ICT and telemedicine has dramatically increased for the management of dermatologic patients [76].

Educational interventions should also be directed at improving adherence to therapy and the utility of distraction techniques for the itch. A variety of educational interventions are possible. These can include face-to-face education in the clinic and workshops, online materials, and social media [77]. A family dealing with a chronic disease, including AD, demands a multidisciplinary approach, compromising bio-pharmacological, educational and instructive, and psychological support. Therapeutic education, as defined by the World Health Organization (WHO) [78], provides not only technical information about the disease and corresponding treatments but also a customized plan developed in partnership with the patient and the patient's caregivers [79].

Recently, medical research on the management of AD has moved from biomedical, technical, and paternalistic patterns to a biopsychosocial and educational model [56]. There is limited awareness and utilization of validated diagnostic criteria in Saudi's routine practice. Thus, awareness campaigns should be promoted to target the knowledge of primary care physicians and pediatricians about the diagnostic criteria for AD. Further research should focus on evaluating the epidemiology, risk factors, and diagnostic pathways for AD in the Kingdom, as well as patients' responses to treatment. The absence of high-quality data indicates the need for more country-based research investigating the awareness, treatment, adherence, and control of symptoms among AD patients in Saudi Arabia. Future studies should evaluate the reasons behind the delayed diagnosis of pediatric AD in Saudi Arabia and primary care physicians' preparedness to deal with AD patients [80, 81].

### 4.4. Impact of AD on Caregivers

The experts agreed that caring for children with AD is time-consuming, and can impact personal relationships, reduce psychosocial functioning, cause sleep impairment, and absence from work among families of affected patients. A previous cross-sectional study reported that caregivers of children with AD had sleep impairment, exhaustion, and social isolation [82]. Similar results were reported by another study where pediatric nurses acknowledged sleep disturbance in parents of affected children with AD which negatively affected the whole family [83]. Therefore, early intervention and psychotherapy are recommended to address these QoL impairments in AD.

Physicians need to specifically ask about QoL impairments to fully understand the toll that AD takes on patients and their families. Family QoL instruments, such as the shortened 10-question Dermatitis Family Impact Questionnaire, can be used to evaluate these effects when available [84]. A cross-sectional study by Shobaili was conducted in Qassim Region of Saudi Arabia from April to July 2009, including 47 children with AD. The parents of children with AD were interviewed via a validated “Dermatitis Family Impact Questionnaire.” Fifteen participants (3.4%) had normal QoL, 104 (23.3%) were mildly affected, 297 (66.4%) were moderately affected, while 31 (6.9%) reported severe impairment in their quality of life. Sleep, monthly expenditure, and food preparation were the highest affected activities. In addition, QoL disturbance was significantly associated with the increasing severity of the disease [85].

In the multidisciplinary approach regarding severe patients, psychiatrists may be involved to provide therapy and education on parenting strategies to help caregivers. Also, nonprofitable organizations and dermatological societies can play a role in providing psychological support and education to help caregivers. The ideal model of intervention integrates different theoretical and operational models, with the participation of a multidisciplinary team composed of the specialist physician (pediatrician, allergist, and dermatologist), the psychologist/psychotherapist, and other professionals, such as nurses [56, 86]. The specialist works as the expert in therapeutic education and first conducts a psychologically supportive educational interview, including evaluation of symptoms, such as itching and sleep disturbance, by quantitative tools, such as the Patient-Oriented SCORAD [87] and a Visual Analogue Scale of Pruritus and approaches for handling itching and sleep impairment [88, 89]. The clinical assessment includes some psycho-diagnostic testing for parents and patients older than 4 years, which helps identify the most useful psychotherapeutic intervention. Psychotherapeutic intervention, provided by a professional expert, supports the patient and family in coping with the emotional pain associated with the disease, improves the stability of their existential and social life, and ultimately improves adherence to treatment [56].

### 4.5. Patient Journey and Criteria for Early Referral

The experts agreed that a large proportion of AD patients in Saudi Arabia are managed directly by primary care physicians and pediatricians. Thus, knowledge of management guidelines, appropriate use of laboratory testing, and proper specialist referrals are crucial for primary care and pediatric physicians. The experts agreed that the percentage of misdiagnoses is approximately 10%. Also, there is a delay in the diagnosis of AD due to several factors, such as insufficient knowledge among general practitioners and lack of MiTime available for using assessment scores. The experts stated that the average duration for delayed diagnosis is around 4–6 months. However, if it is severe eczema, patients are referred in 2 or 3 weeks. In mild patients, they are usually treated with topical steroids which are available at the dispensary level.

In Saudi Arabia, only a small portion of moderate-to-severe AD patients are referred early to dermatology clinics. There is limited practical knowledge among healthcare providers and general practitioners about the early referral of severe pediatric AD patients [90]. An experienced dermatologist should be involved in the proper diagnosis and treatment of severe patients with AD and those who are resistant to standard treatment, as other skin disorders such as some genodermatoses, scabies, psoriasis, and cutaneous T cell lymphoma may be considered for differential diagnosis [91]. Specific attention should be given to children with severe AD as they are more likely to persist into adulthood [92, 93].

**Figure 3:**
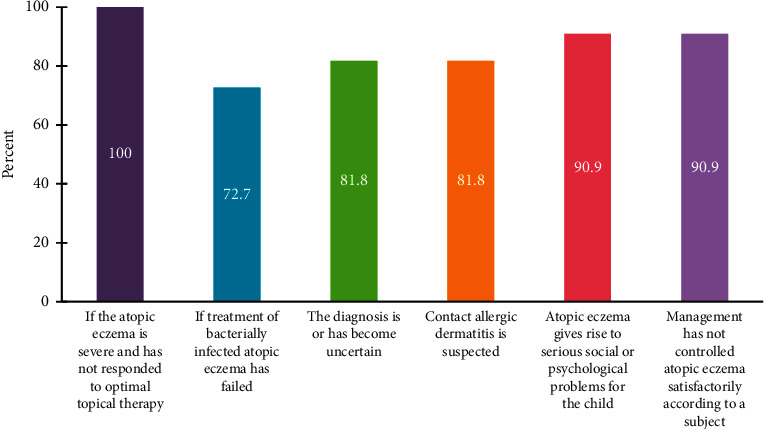
Reasons for referral of pediatric patients with AD.

A guideline for general practitioners for referral criteria and timeline is required to ease the process of referral. General practitioners are encouraged to refer children with AD who have a family history of atopy, and upon the presence of the following conditions: severe atopic eczema that has not responded to optimal topical therapy, failure of bacterially infected ectopic eczema, uncertain diagnosis, suspected contact allergic dermatitis, and serious social or psychological problems caused by AD [94–96] (Figure 3). The experts agree that pediatricians should urgently refer AD patients if eczema herpeticum is suspected. Eczema herpeticum is an emergency in dermatology, mainly in children aged less than 2 years, that requires urgent referral to a proper pediatric institution for review and management. Eczema herpeticum can cause severe sequelae, including eye or meningeal involvement leading to scarring [97].

**Figure 4:**
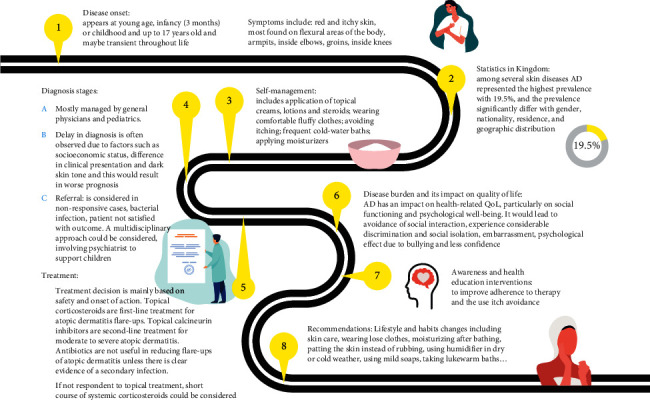
Roadmap of pediatrics AD patient in Saudi Arabia.

The referral system somehow varies among different hospitals of the Ministry of Health. The electronic referral system proved to be of great help. Fortunately, Saudi Arabia has an easy and quick referral system for dermatologists [98, 99]. Hurdles of pediatric AD patient journey in Saudi Arabia are shown in Figure 4.

## 5. Conclusion

Pediatricians and dermatologists commonly encounter AD; however, it is still under-recognized in its early stage in Saudi Arabia. The use of available disease severity scales and QoL scale is limited in routine Saudi practice, owing to their complexity and time-consuming nature. The present consensus combined the best available evidence and clinical experience to optimize the diagnosis and management of AD in Saudi Arabia. The experts developed several statements to aid primary care physicians, dermatologists, pediatricians, and family physicians in diagnosing and managing AD presenting to primary and advanced healthcare settings in Saudi Arabia. The treatment of pediatric AD should be based on shared decision-making between the parents/caregivers and different specialties. Proper assessment of severity and treatment with emollients should be recommended. Multidisciplinary collaboration is needed to develop a national guideline covering all stakeholders to share their ideas and suggestions.

## Figures and Tables

**Table 1:** Consensus statements developed and consensus percentage obtained.

Domain	Statement	Consensus percentage (%)
The diagnosis and severity assessment of AD	(1) AD is commonly encountered by pediatricians and dermatologists; nonetheless, it is still under-recognized in its early stage in Saudi Arabia. Thus, an expert dermatologist should be involved in the assessment of suspected children with a family history of atopy, particularly in patients with isolated lesions	100
	(2) AD diagnosis should be documented, showing the criteria used, key morphological characteristics, and features used to ascertain the severity of the disease. Nonetheless, there is still a need for simple validated diagnostic criteria for pediatric AD suitable for daily practice	100
	(3) The use of available disease severity scales and QoL scale is under-utilized in routine Saudi practice, owing to their complexity and time-consuming nature. Although global assessment scores have not been validated in office settings, they can be used for the simple classification of AD severity	100
	(4) Clinicians should examine the impact of disease on QoL during clinic visits. There is a need for validated Arabia patient-reported outcomes to measure the impact of AD on the QoL of the patients and their parents/caregivers	100
	(5) Although lab tests and biomarkers can be used initially in the diagnosis of AD, there is no need for biomarkers for assessing the severity of AD	75
	(6) Although AD is primarily a clinical diagnosis, many unrequired tests are usually ordered by the healthcare providers in the Saudi setting, despite the lack of sufficient data to support their diagnostic and/or prognostic utility	100
	(7) The development and utilization of severity scores, with validated thresholds for treatment choice, are critical steps as the treatment's decision is mainly based on disease severity	92
AD treatment	(8) There is a discrepancy between dermatologists, pediatricians, allergists, immunologists, and family physicians in managing atopic dermatitis in children	92
	(9) The treatment of pediatric AD should be based on shared decision-making between the parents/caregivers and the provider. Educational programs for parents and health care providers are an important element of shared decision-making	100
	(10) The shared decision-making should involve the following:	
	(i) Treatment goals and expectations	100
	(ii) Strategy planned to reach these goals	91
	(iii) Therapeutic options	100
	(iv) Risks and benefits	100
	(v) The impact of associated comorbidities	91
	(vi) Parents' preference	92
	(11) In the local Saudi setting, therapeutic protocols are still lacking. These protocols should incorporate	
	(i) Therapeutic goals (endpoints, time points)	100
	(ii) Criteria for eligibility for topical therapies, including nonsteroidal topical therapies	91
	(iii) Criteria for monitoring response to systemic treatments	100
	(12) The long-term management of pediatric AD is still challenging, owing to the variability in efficacy of available therapies in different patients' profiles and the remittent-relapsing course of the disease	100
	(13) Severe AD in children is likely to persist in adolescence and adulthood	
	A long-term curative strategy including patient education, trigger avoidance, proper skincare, and compliance to pharmacologic therapies and nonpharmacologic measures is essential	100
	(14) Many children with moderate-to-severe AD are not receiving systemic therapy because of a lack of recommendations concerning indication and appropriate timing of systemic treatments	
	The introduction of systemic therapies is usually delayed, which impacts the response to therapy	91
	(15) Systemic steroids are effective but are associated with unacceptable short- and long-term adverse events and, therefore, should be used with caution and in very limited circumstances for severe exacerbations for a short course	100
	(16) Because of safety concerns, many immunosuppressive treatments (such as azathioprine, cyclosporine, and methotrexate) are not recommended for long-term use in children with AD. Insufficient data exist to make clear recommendations regarding the optimal immunosuppressants dosing or duration	91
	(17) The limited number of approved therapies does not allow the development of a therapeutic algorithm	92
	(18) The introduction of new biologic therapies will likely allow for improved treatment of pediatric AD and attempt to address the unmet needs of AD treatment in this population	91
	(19) Real-world data and local experience with new biologic therapies are necessary to evidently support the use of biologics for indicated patients	91
	(20) A considerable proportion of children with AD in Saudi Arabia do not adequately comply with the prescribed treatment. As most treatments are administrated at home with little hospital services involvement, there remain significant challenges in ensuring optimal treatment compliance	91
	(21) The chronicity and necessity for multiple treatment vehicles may add to the complexity of treatment and barriers to adherence	100
	(22) In Saudi Arabia, parents' concerns over the safety profiles of topical and systemic therapies can lead to compliance issues and treatment delays or restrictions	91
	(23) Corticophobia is a real issue in Saudi Arabia and needs to be promptly recognized and overcome by patient education programs	100
	(24) Other factors that contribute to limited compliance in Saudi Arabia include the financial burden of long-term treatment and limited awareness among parents/caregivers	82
	(25) Various strategies should be examined to improve adherence to topical treatment, such as telemedicine technology and proactive intermittent treatment strategies	91
	(26) There is a need to develop unified validated criteria for assessing treatment response in pediatric AD, as there are no accepted criteria for defining treatment failure	100
	(27) There is no consensus regarding the optimal treatment duration to demonstrate the efficacy of topical treatments for AD	91
	(28) There are currently no acceptable biomarkers that would predict response to treatment in the setting of pediatric AD	100
Education and research of AD	(29) Educational measures are critical components of any treatment strategy for AD. These measures should be tailored according to patient- and disease-specific factors. Possible educational tools include using traditional materials, support groups, and mobile apps	100
	(30) Educational interventions should also be directed at improving adherence to therapy and the utility of distraction techniques for the itch. A variety of educational interventions are possible. These can include face-to-face education in the clinic and workshops, online materials, and social media	100
	(31) The education measures should aim to inform parents/caregivers about symptoms and signs of bacterial infection of AD: weeping, pustules, crusts, eczema failing to respond to treatment, rapidly worsening eczema, fever, and malaise	91
	(32) Written care plans should cover treatment of flares and episodes of infected eczema to educate parents/caregivers on when topical corticosteroids (and other treatments) are appropriate	100
	(33) In Saudi's routine practice, there is limited awareness and utilization of validated diagnostic criteria. Thus, awareness campaigns should be promoted to target the knowledge of primary care providers and pediatricians about the diagnostic criteria for AD	100
	(34) There is a limited number of published literature that investigates pediatric AD in Saudi Arabia. Future studies should focus on evaluating the epidemiology, risk factors, and diagnostic pathways for AD in the kingdom, as well as patients' responses to treatment	100
	(35) The absence of high-quality data indicates the need for more country-based research investigating the awareness, treatment, adherence, and control of symptoms amongst AD patients in Saudi Arabia	100
	(36) Future studies should evaluate the reasons behind the delayed diagnosis of pediatric AD in Saudi Arabia and primary care physicians' preparedness to deal with AD patients	100

Impact of AD on caregivers	(37) Caring for children affected by AD can be an extremely time-consuming task that can impair personal relationships, decrease psychosocial functioning, cause sleep loss, and absence from work among family members of affected patients. Early intervention and psychotherapy are recommended to address these QoL impairments AD	91
	(38) Physicians need to specifically ask about QoL impairments to fully understand the toll that AD takes on patients and their families. Family QoL instruments, such as the shortened 10-question dermatitis family impact questionnaire, can be used to evaluate these effects when available	91
	(39) In the multidisciplinary approach with severe patients psychiatrists may be involved to provide therapy and education on parenting strategies to help caregivers	92
	(40) Nonprofitable organizations and dermatological societies can play a role in providing psychological support and education to help caregivers	100
	(41) Multidisciplinary education programs are needed to provide education and support for caregivers of patients with AD	91

Patient journey and criteria for early referral	(42) A large proportion of AD patients in Saudi Arabia are managed directly by primary care physicians and pediatricians. Thus, knowledge of management guidelines, appropriate use of laboratory testing, and proper specialist referrals are crucial	100
	(43) In Saudi Arabia, only a small portion of AD patients are referred early to dermatology clinics. There is limited practical knowledge among healthcare providers and general practitioners about the early referral of pediatric AD patients	92
	(44) Typically, pediatric patients with AD are referred only upon severe diseases and no response to topical treatment. Early referral to a skilled dermatologist may improve the outcome of the patients before the development of severe AD	100
	(45) In Saudi Arabia, healthcare providers are encouraged to refer children with allergic dermatitis, who had a family history of atopy, upon the presence of the following:	
	(i) If the atopic eczema is severe and has not responded to optimal topical therapy	100
	(ii) If treatment of bacterially infected ectopic eczema has failed	73
	(iii) The diagnosis is or has become uncertain	82
	(iv) Contact allergic dermatitis is suspected	82
	(v) Atopic eczema gives rise to serious social or psychological problems for the child	91
	(vi) Management has not controlled atopic eczema satisfactory according to a subject	91
	(46) Pediatricians should urgently refer AD patients if eczema herpeticum is suspected	91

**Table 2:** Major and minor criteria for diagnosis of AD according to Hanifin and Rajka.

Major features (3 of 4 required)
(1) Pruritus
(2) Typical morphology and distribution
(2.1) flexural lichenification or linearity in adults
(2.2) facial and extensor involvement in infants and children
(3) Chronic or chronically relapsing dermatitis
(4) Personal or family history of atopy, such as asthma, allergic rhinitis, and atopic dermatitis
Minor features (3 of 23 required)
(1) Xerosis
(2) Ichthyosis/palmar hyperlinearity/keratosis pilaris
(3) Immediate (type 1) skin test reactivity
(4) Elevated serum immunoglobulin E
(5) Early age of onset
(6) Tendency toward cutaneous infections (*S. aureus* and herpes simplex virus)/impaired, cell-mediated immunity
(7) Tendency toward nonspecific hand or foot dermatitis
(8) Nipple eczema
(9) Chelitis
(10) Recurrent conjunctivitis
(11) Dennie-morgan infraorbital fold
(12) Keratoconus
(13) Anterior subcapsular cataract
(14) Orbital darkening
(15) Facial pallor/facial erythema
(16) Pityriasis alba
(17) Anterior neck fold
(18) Itch when sweating
(19) Intolerance to wool and lipid solvents
(20) Perifollicular accentuation
(21) Food intolerance
(22) Course influence by environmental/emotional factors
(23) White dermographism/delayed blanch

## Data Availability

Data will be shared when requested.
